# Function and Evolution of the Plant MES Family of Methylesterases

**DOI:** 10.3390/plants13233364

**Published:** 2024-11-29

**Authors:** Timothy A. Chaffin, Weijiao Wang, Jin-Gui Chen, Feng Chen

**Affiliations:** 1 Graduate School of Genome Science and Technology, University of Tennessee, Knoxville, TN 37996, USA; tchaffi2@utk.edu; 2 Department of Plant Sciences, University of Tennessee, Knoxville, TN 37996, USA; wwang82@vols.utk.edu; 3 Biosciences Division, Oak Ridge National Laboratory, Oak Ridge, TN 37831, USA; chenj@ornl.gov; 4 The Center for Bioenergy Innovation, Oak Ridge National Laboratory, Oak Ridge, TN 37831, USA

**Keywords:** demethylation, α/β hydrolase, methyl esters, defense

## Abstract

Land plant evolution has been marked by numerous genetic innovations, including novel catalytic reactions. Plants produce various carboxyl methyl esters using carboxylic acids as substrates, both of which are involved in diverse biological processes. The biosynthesis of methyl esters is catalyzed by SABATH methyltransferases, and understanding of this family has broadened in recent years. Meanwhile, the enzymes catalyzing demethylation—known as methylesterases (MESs)—have received less attention. Here, we present a comprehensive review of the plant MES family, focusing on known biochemical and biological functions, and evolution in the plant kingdom. Thirty-two *MES* genes have been biochemically characterized, with substrates including methyl esters of plant hormones and several other specialized metabolites. One characterized member demonstrates non-esterase activity, indicating functional diversity in this family. *MES* genes regulate biological processes, including biotic and abiotic defense, as well as germination and root development. While *MES* genes are absent in green algae, they are ubiquitous among the land plants analyzed. Extant *MES* genes belong to three groups of deep origin, implying ancient gene duplication and functional divergence. Two of these groups have yet to have any characterized members. Much remains to be uncovered about the enzymatic functions, biological roles, and evolution of the MES family.

## 1. Introduction to the Methylesterase Family

Methylation and demethylation are opposing biochemical reactions that occur in DNA, proteins, polysaccharides, and diverse small-molecule metabolites [1,2]. As such, they are important in many biological processes [3,4,5]. Among the small-molecule reactants and products of these reactions in plants are carboxylic acids and their respective methyl esters [6,7]. One protein family that catalyzes the methylation of carboxylic acids to form methyl esters is the SABATH methyltransferase family [4]. The known substrates of the SABATH family include plant hormones such as indole-3-acetic acid (IAA), gibberellic acid (GA), salicylic acid (SA), and jasmonic acid (JA). Methylation of these important metabolites regulates their availability. Their products, the respective methyl esters, may have new biological functions that differ from their substrates [4]. Since the establishment of the SABATH family in 2003, much progress has been made in discovering new catalytic and biological functions of *SABATH* genes [4,8]. Equally exciting was the discovery of the enzyme family that catalyzes the reverse reaction, namely demethylation of carboxylic acid methyl esters. This new protein family has been denoted the methylesterase (MES) family [9]. In this article, we provide a comprehensive synthesis of the studies of the MES family, especially their biochemical and biological functions. We also performed phylogenetic analysis to understand the evolution of the MES family in land plants.

## 2. MES Family History: Discovery and Relatedness to Other Enzyme Families

The first member of the plant MES family to be identified was polyneuridine aldehyde esterase (PNAE) from *Rauvolfia serpentina*, with the gene being reported in 2000 [10]. Following this, a gene encoding a methyl jasmonate esterase was discovered in tomato (SlMJE) in 2004 [11]. SlMJE was found to share significant sequence homology with PNAE [11]. The breakthrough discovery came with the realization that salicylic acid binding protein 2 from *Nicotiana tabacum* (NtSABP2) functions as methyl salicylate esterase [12]. It was found that NtSABP2, SlMJE, and PNAE all belong to the same protein family [12]. The name of the methylesterase (MES) family was coined when this protein family was investigated in *Arabidopsis thaliana* [9], to distinguish it from other families of similar function.

While the MES family was named to separate it from other families, this name can also lead to confusion with other methylesterases. Pectin methylesterases (PMEs) are a separate family of enzymes that have esterase activity with methylated pectin subunits, allowing them to alter pectin structure [5,13]. PMEs are prominent in plants but also exist in fungi and bacteria, especially in plant pathogens [5]. Meanwhile, the MES family is currently limited to the plant kingdom. Although they share the methylesterase name, MESs are not closely related to PMEs. They have distinct structures and catalytic mechanisms, leading to their respective esterase activities [13]. PMEs belong to the carbohydrate esterase family, while MESs are a part of the α/β hydrolase superfamily, one of the largest groups of enzymes that exist in all domains of life [13,14].

Members of the α/β hydrolase superfamily share a conserved catalytic triad, typically composed of either Ser-His-Asp or Ser-His-Glu [15,16]. Most members also share an oxyanion hole, which is known to stabilize reaction intermediates [16]. Despite these shared structures, members of the α/β hydrolase superfamily have diverse origins and functions [17]. Carboxylesterases (CXEs), another group within this superfamily, are also responsible for hydrolysis of carboxylesters [7]. Previously, the MES family was classified as a subcategory of CXE due to functional similarity between these groups [7]. Later, other studies showed that MESs are phylogenetically distinct from CXEs, indicating that they are not part of the same evolutionary lineage [9,18]. Furthermore, CXE enzymes can utilize a wide range of ester substrates [19,20]. MES members, on the other hand, are more limited in their known substrates, with most utilizing methyl esters of important plant hormones [9,11,21]. However, enzymatic diversity exists in this family, even among currently characterized members (Table 1). In the next section, we will describe in detail the known biochemical functions of the MES family.

## 3. Known Enzymatic Functions of MES Members

**Figure 1:**
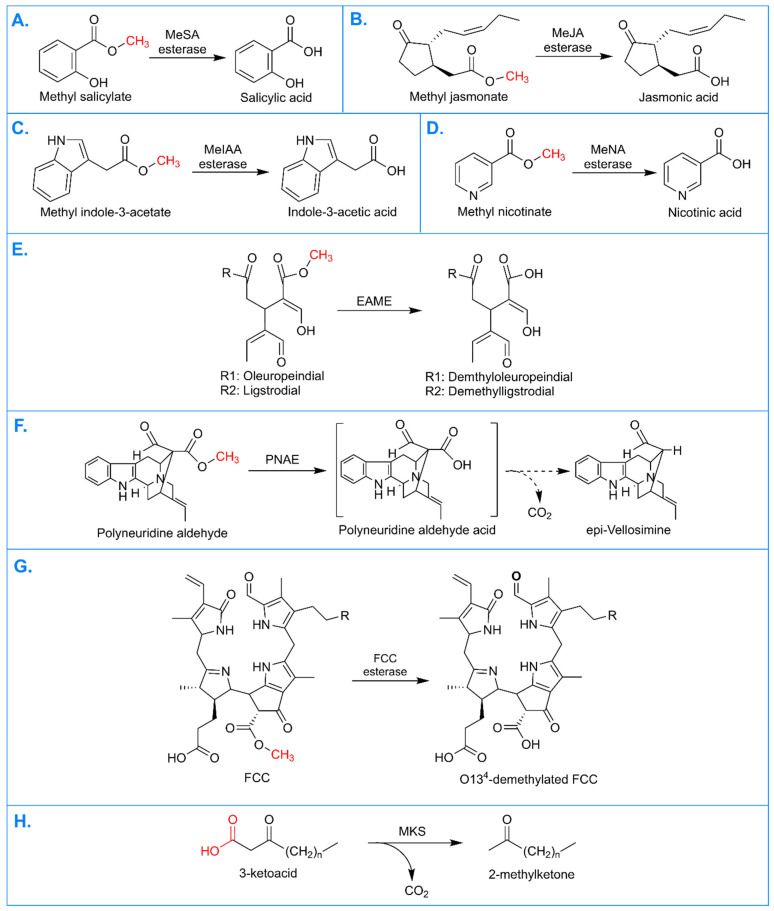
Biochemical reactions catalyzed by MES enzymes. The site of reaction for each substrate is indicated in red. (**A**) Reaction catalyzed by methyl salicylate (MeSA) esterase. (**B**) Reaction catalyzed by methyl jasmonate (MeJA) esterase. (**C**) Reaction catalyzed by methyl indole-3-acetate (MeIAA) esterase. (**D**) Reaction catalyzed by methyl nicotinate (MeNA) esterase. (**E**) Reaction catalyzed by elenolic acid methylesterase (EAME). (**F**) Reaction catalyzed by polyneuridine aldehyde esterase (PNAE). (**G**) Reaction catalyzed by fluorescent chlorophyll catabolite (FCC) esterase. (**H**) Reaction catalyzed by methyl ketone synthase (MKS).

MES enzymes are currently known to utilize three methylated plant hormones as substrates, including methyl esters of SA, JA, and IAA (Figure 1A–C). Methyl salicylate (MeSA) esterases are by far the most represented among characterized members, with 18 known enzymes utilizing MeSA as their major substrate (Table 1). This class of enzymes—including NtSABP2—demethylates MeSA into SA, which is known to be crucial for signaling pathways [40]. Similarly, methyl jasmonate esterases (MJEs) demethylate methyl jasmonate (MeJA) into JA, another important signaling hormone [41]. Eight MES members have been found to have MJE activity (Table 1). Finally, three genes are identified as having esterase activity with methyl IAA (MeIAA) (Table 1). These genes demethylate MeIAA into IAA, one of the most well-studied plant hormones [42]. It is important to note that several genes show in vitro activity with multiple hormone substrates (Table 1). This highlights the importance of in vivo testing for determining the true biological function of MES enzymes.

MES enzymes can also favor substrates that are not hormones but maintain structural similarity to hormones. AtMES2 from Arabidopsis is known to utilize methyl nicotinate (MeNA) as a substrate, the structure of which is nearly identical to that of MeSA (Figure 1D) [37]. Demethylation of MeNA allows the recycling of nicotinate (NA) for an alternative biosynthesis pathway to maintain nicotinamide adenine dinucleotide (NAD) levels [37]. This is a crucial function in all plants; thus, it is likely that homologs of AtMES2 may be found across plant taxa.

Some MES members are also known to utilize specialized products to generate secondary metabolites. This includes elenolic acid methylesterases 1 and 2 (EAME1 and EAME2), which utilize secoiridoids as their substrates, including oleuropeindial and ligstrodial (Figure 1E). These enzymes are involved in the production of secoiridoid-derived polyphenols such as oleuropein [36]. Intriguingly, researchers also found that EAME1 and EAME2 can convert MeJA and MeIAA into ethyl JA and ethyl IAA, respectively, in the presence of ethanol [36]. This transesterification capability is not currently known to exist in any other MES enzymes. It is unclear whether this in vitro activity mirrors a true native function, but it indicates an interesting area of further study for novel MES capabilities. PNAE, the first characterized MES member, utilizes polyneuridine aldehyde as its substrate, which is structurally related to secoiridoids. PNAE converts polyneuridine aldehyde into polyneuridine aldehyde acid, an intermediate that is then nonenzymatically converted to epi-vellosimine (Figure 1F). This leads to downstream production of ajmaline, a compound of interest from *Rauvolfia serpentina* [10].

MES reactions with more complex substrates are also possible. AtMES16 from Arabidopsis has been found to break down fluorescent chlorophyll catabolites (FCCs) in the chloroplast, an important step in chlorophyll degradation [38]. AtMES16 was shown to demethylate FCCs at oxygen O13^4^, generating O13^4^-demethylated FCCs (Figure 1G). This demethylation was shown to be important for subsequent degradation steps [38]. FCCs are currently the largest known substrate of an MES enzyme, demonstrating the possibilities of this family. Chlorophyll degradation is important in all plants, though there are differences in the specific steps of FCC breakdown [38]. Thus, homologs of AtMES16 are likely to be found in other species but may not be conserved across all taxa. For example, the radish enzyme RsPPD has been shown to have similar functionality and, furthermore, shares sequence similarity with AtMES16 [38,43]. RsPPD has not been confirmed as a member of MES, but further study of this enzyme and other possible AtMES16 homologs can expand the understanding of specialized MES functions.

Finally, the enzyme ShMKS1 from the wild tomato species *Solanum habrochaites* has been found to be involved in methylketone synthesis [39]. Notably, the typical Ser-His-Asp triad is not found in this enzyme but is replaced by an Ala-His-Asn triad [44]. This change leads to unique enzymatic activity compared to other MES members. ShMKS1 catalyzes the final step of methylketone biosynthesis by converting 3-keto acids to 2-methylketones through decarboxylation (Figure 1H). This demonstrates that some MES members may have evolved new, non-esterase functions. Consideration of currently known MES reactions is useful in identifying activities of new members. Equally helpful is an understanding of MES biological functions, which will be discussed in the following section.

## 4. MES Biological Functions: Defense and Development

**Figure 2:**
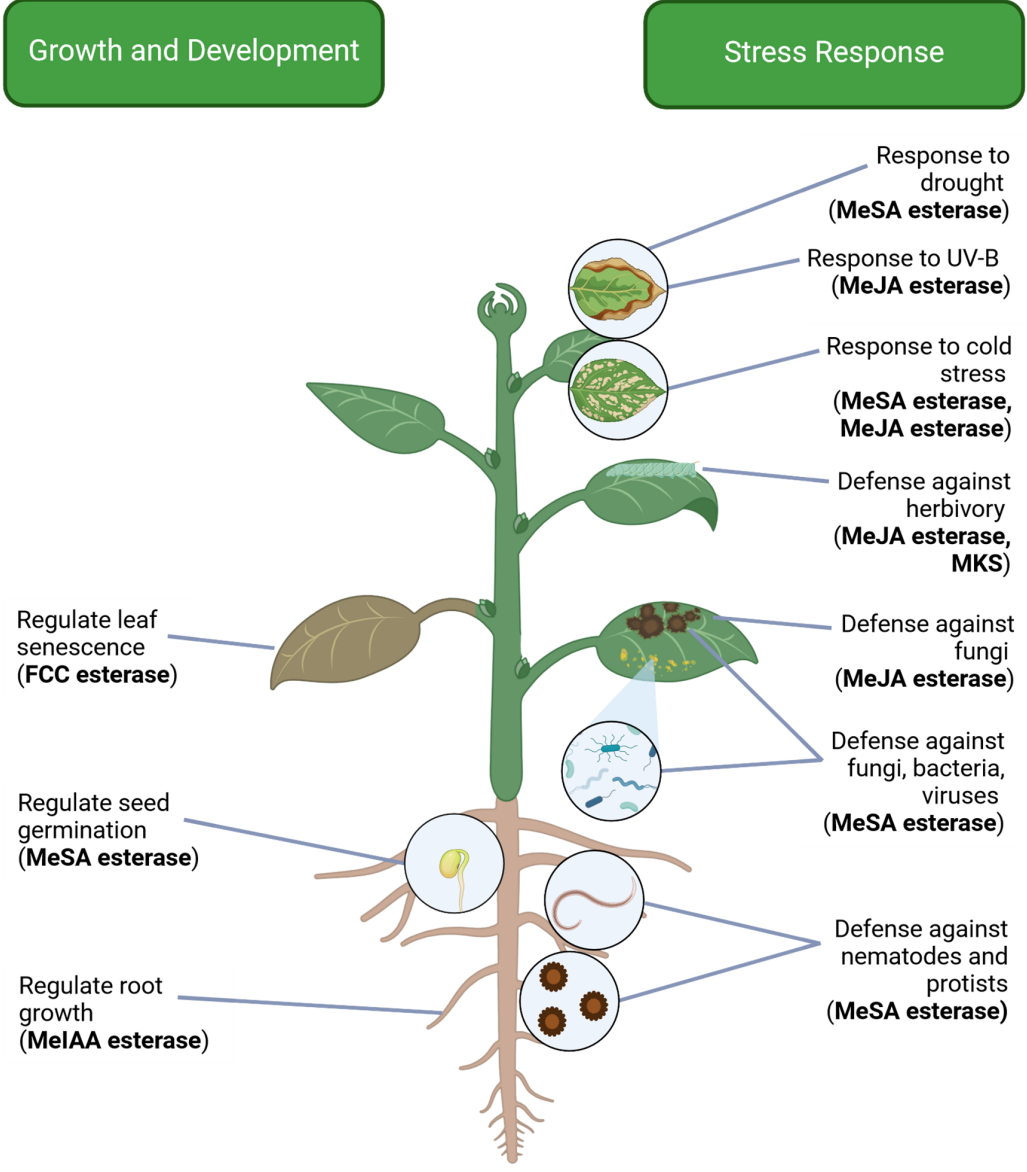
Known biological functions of MES enzymes in plants based on in vivo genetic studies. Created with BioRender. https://BioRender.com/v52h282 (accessed on 12 November 2024).

The majority of known MES members demethylate a specific phytohormone, which is important for the regulation of hormone concentrations and their respective processes. However, other MES members are involved in enzymatic reactions with other plant compounds, which can have a variety of functions throughout plant tissues and life stages. Currently, most biologically characterized *MES* genes are found to be involved in the stress response, though a few also factor into developmental processes (Figure 2). Understanding what is known about the biological roles of MES enzymes can lead to better knowledge of their origins.

### 4.1. MeSA Esterases: Pathogen Defense, Drought Response, and Seed Germination

Being sessile organisms, plants must rely on chemical signaling pathways to defend themselves against stress and pathogens. The plant hormone salicylic acid (SA) has long been known to be crucial for defense against viral, bacterial, and fungal pathogens through the systemic acquired resistance (SAR) pathway [45,46,47]. Methylation of SA into MeSA is a crucial part of this signaling process [40]. Because MeSA can be transported more easily and become airborne, it serves as a mobile form to induce resistance pathways in uninfected tissues as well as neighboring plants [48]. Methylation of SA is performed by SAMTs and BSMTs, well-known members of the SABATH family [49,50]. However, once MeSA arrives in its target tissues, it must be demethylated back into SA to induce the SAR pathway.

Following its initial discovery, NtSABP2 was found to be involved in disease resistance by catalyzing the conversion of MeSA into SA [12,21]. Confirming the importance of MeSA in SAR signaling, it was shown that overexpression of *NtSABP2* leads to reduced MeSA and impaired SAR infection response [40]. However, in a more recent study, overexpressing *NtSABP2* in transgenic citrus fruit was found to enhance resistance to bacterial infection [51]. This is in line with research on *SAMT* genes, where modulation of SA:MeSA ratios can either hamper or enhance disease resistance in different cases [52,53]. This highlights the importance of understanding these genes and their involvement in immune pathways to correctly predict resulting phenotypes.

The *NtSABP2* sequence has also been used to identify various *MES* genes in other species, many of which have been confirmed to have MeSA esterase activity (Table 1). Several of these have also been demonstrated to be directly involved in pathogen response(Figure 2). *AtMES1*, *-7*, and -*9* from Arabidopsis were shown to be activated in response to infection with *Pseudomonas syringae*, and knockdown of these genes leads to inhibition of SAR pathways [22]. Similarly, CsMES1 from sweet orange was shown to be important for protection against citrus canker. *CsMES1* transcripts were upregulated during pathogen infection; meanwhile, inhibition of this gene leads to increased canker formation [25]. In addition to bacterial pathogens, SA is also involved in responses to fungal pathogens. StMES1 from potato was found to be necessary for the SAR response to the potato blight fungus *Phytophthora infestans* [32]. Suppression of *StMES1* led to compromised SAR responses against the fungus. PvMES1 from the common bean has similarly been shown to control SA signaling in response to *Fusarium oxysporum* [30]. It was found that overexpression of *PvMES1* enhanced resistance, while silencing it led to increased susceptibility [30]. Likewise, FvMES2 from strawberry is an MeSA esterase involved in the fungal response. The gene was found to be upregulated in response to *Botrytis cinerea* infection, which was also highly correlated with SA signaling [26]. Meanwhile, strawberries overexpressing *FvMES2* show increased resistance to *B. cinerea* [26]. MeSA esterases are also involved in specialized infection responses to protists and parasitic animals. The *Brassica napus* member BnMES34 is an MeSA esterase that controls responses to the protist pathogen *Plasmodiophora brassicae* [24]. It was found that heterologous expression of *BnMES34* in Arabidopsis confers resistance to the clubroot disease caused by *P. brassicae* [24]. Finally, MeSA esterases can also respond to infection by nematodes. GmSABP2-1 from soybean was recently found to have MeSA activity, which can confer enhanced resistance to soybean cyst nematode [27]. This is in line with previous research in SAMTs, confirming that MeSA regulation is important during nematode infection [54].

Although pathogen response is perhaps the most discussed role of SA, it is not the only function of this hormone. Supporting this, some MeSA esterases have been found to have activities in other areas of stress response and plant development (Figure 2). LcSABP, an NtSABP2 ortholog from *Lycium chinense*, was found to enhance drought tolerance in transgenic tobacco [28]. It was found that this tolerance was conferred through an SA-dependent pathway, leading to increased production of reactive oxygen species and stress-responsive genes [28]. Similarly, AtMES9 was recently found to support an SA-mediated pathway during cold stress conditions [55]. Cold stress was shown to upregulate *AtMES9* and other SA biosynthesis genes to maintain SA concentrations [55]. In another example of diverse regulation, AtMES7 was shown to modulate seed germination in Arabidopsis [23]. AtMES7 controls seed SA levels under normal and salt stress conditions, leading to varied germination responses [23]. As noted previously, *AtMES7* and *-9* were originally shown to activate expression in response to pathogen infection [22]. The more recent analyses demonstrate the possibility of *MES* genes regulating multiple processes at different times or in diverse tissues. This highlights the importance of in-depth biological function determination. Many MeSA-utilizing MES enzymes are known, but their functions are not necessarily limited to SAR pathogen response. Studying these members under the lens of other processes, such as abiotic stress or plant development, can lead to a better understanding of their in planta biological roles.

### 4.2. MeJA Esterases: Biotic and Abiotic Stress Response

Jasmonic acid (JA) is an established plant hormone that plays roles in an array of plant processes, including germination, growth, fruit ripening, and resistance to biotic and abiotic stresses [41,56,57]. Methyl jasmonate (MeJA) is a derivative of JA and is produced by JAMT of the SABATH family [58]. Similar to MeSA, MeJA serves as a mobile transport form to elicit responses throughout the plant or to neighboring plants [59,60]. MeJA is also considered an inactive form and must be demethylated back to JA for the regulation of biological processes [34]. This requires the action of an MeJA esterase (MJE), which is part of the MES family. These enzymes can serve various biological roles, notably in response to biotic and abiotic stresses (Figure 2).

SlMJE1, the first categorized MJE, was discovered in tomato and has been found to be important for the response to fungal pathogens [11,61]. Interestingly, neither overexpression nor RNAi knockdown of *SlMJE1* shows consistent changes in levels of MeJA or other jasmonate derivatives [61]. Despite this, both conditions lead to increased susceptibility to the fungal pathogen *Sclerotinia sclerotiorum* [61]. Further study of this gene can help clarify how it regulates plant response to the fungus. On the other hand, JA is particularly known for its role in response to herbivory. NaMJE from *Nicotiana attenuata* is known to be involved in insect resistance. It was found that the conversion of MeJA back to JA is specifically required for resistance to the tobacco hornworm *Manduca sexta* [34]. In addition to biotic stress, JA signaling can also be involved in the abiotic stress response [56]. VvMJE1 from grapevine was shown to have MJE activity specifically activated by abiotic stresses, including cold stress and UV-B treatment [35]. Because JA can be involved in various plant responses, it is important to characterize the biological functions of MJE members. Several other enzymes with MJE activity have been identified, but their in vivo functions remain unknown (Table 1).

### 4.3. MeIAA Esterases: Regulation of Auxin-Mediated Development

Auxins are a class of plant hormones that are crucial for proper growth and development. Indole-3-acetic acid (IAA), the most common auxin, regulates diverse processes including cell elongation and differentiation, organ development, and phototropism [42,62]. Plants are known to utilize various IAA conjugates to regulate auxin levels, some of which are considered irreversible intermediates in auxin degradation [62,63]. Methyl IAA (MeIAA), on the other hand, has been used in experiments as a surrogate for IAA for many years, and is now known to be reversible to IAA through the action of an MES enzyme in Arabidopsis, AtMES17 [9,64]. It was found that *AtMES17* mutants had altered responses to exogenous MeIAA—but not exogenous IAA—in regulating root growth [9] (Figure 2). This demonstrates the importance of demethylating MeIAA for proper auxin regulation. It is likely that this enzyme could regulate IAA in other parts of the plant as well, but this requires further study. Additionally, AtMES17 is the only enzyme that has been experimentally characterized as an MeIAA esterase in vivo, though other putative MeIAA esterases have been identified (Table 1). A number of recent studies have bioinformatically identified MES members from different species, including possible homologs of AtMES17 [18,24,35]. Whether these are true MeIAA esterases remains an open question. However, due to the importance of IAA, it is likely that MeIAA esterases will be well conserved across plant taxa. Further characterization of AtMES17 and other MeIAA esterases will be necessary to elucidate their native functions.

### 4.4. AtMES2: NAD Recycling

Nicotinamide adenine dinucleotide (NAD) is a crucial coenzyme in plants as well as in other organisms [65,66]. Because of its importance in many diverse processes, it is necessary to maintain adequate levels of NAD throughout the plant. NAD can be synthesized either via a de novo pathway, or it can be recycled from other derivatives [65]. The NAD salvage pathway begins with nicotinate (NA), but NA is known to be toxic to plants at high concentrations [67]. Due to this toxicity, it is converted into various other forms, including methyl nicotinate (MeNA), by an N-methyltransferase [68]. It was recently found that MeNA serves as a long-distance transport form of NA, which can later be demethylated by AtMES2 from Arabidopsis and recycled into NAD [37]. This is reminiscent of the roles of MeSA and MeJA and illustrates one of the reasons that methylated products are common in plants. This recycling process via MeNA can allow plants quick access to NAD, while ensuring that NA toxicity does not cause cellular damage. Furthermore, it is well established that abiotic stresses lead to increased degradation of NAD [65]. Supporting this, it was found that *AtMES2* is suppressed under abiotic stress inducers, indicating that it may be involved in stress adaptation [37]. However, its true biological roles are not yet fully understood. Further study of this gene, as well as identification of homologs in other species, will expand understanding of NAD regulation by MES family members.

### 4.5. AtMES16: Chlorophyll Degradation During Leaf Senescence

Chlorophyll is the most abundant pigment on Earth and is clearly fundamental to plant survival [69]. Despite its importance, chlorophyll can also be toxic to cells and must be degraded during processes such as fruit ripening and leaf senescence [69]. This requires first the conversion of chlorophylls a and b into fluorescent chlorophyll catabolites (FCCs) [70]. These FCCs are then demethylated before passing through subsequent breakdown steps, and this demethylation is performed by AtMES16 in Arabidopsis [38]. This enzyme was shown to be important for chlorophyll degradation during leaf senescence, and mutants were seen to have increased retention of FCCs [38] (Figure 2). AtMES16 may be related to the previously identified radish enzyme RsPPD, which is known to demethylate pheophorbide, another member of the chlorophyll degradation pathway [43]. RsPPD has not been identified as a member of MES, but due to its homology and functional similarity to AtMES16, further investigation of this enzyme as a possible MES member is warranted. Furthermore, it is likely that MES members performing chlorophyll degradation may be found across plant taxa. The study of such proteins may reveal new roles in controlling leaf senescence and fruit ripening.

### 4.6. PNAE, EAME1, EAME2, and ShMKS1: Putative Defense and Stress Responses

PNAE from *Rauvolfia serpentina* was the first MES member to be functionally characterized [10]. Despite many years of study on this enzyme, its native biological functions remain unknown. However, examining the pathways it is involved in may hint at its possible roles. PNAE catalyzes the conversion of polyneuridine aldehyde into epi-vellosimine, an important precursor for ajmaline biosynthesis. Ajmaline is anthropologically significant due to its long history of use as a cardiovascular drug [71]. In plants, ajmaline has been found to be upregulated after treatment with MeJA, indicating a possible role in stress response or other areas of JA signaling [72]. Epi-vellosimine is also a precursor to the sarpagine-type alkaloids, making PNAE a catalyst in this pathway as well. Sarpagines are also thought to serve defensive functions similar to ajmaline and other alkaloids [73,74]. Further study of PNAE may reveal its biological role, perhaps in plant defense or other functions.

EAME1 and EAME2 were identified from olive based on their homology to PNAE and found to be involved in secoiridoid biosynthesis [36]. Oleuropein is the most common secoiridoid in olives, found in high levels in both leaves and fruits [75,76]. It is thought to serve a defense role against herbivory by activating protein denaturation mechanisms and decreasing nutritional value upon tissue damage [77]. Similar to PNAE, the true biological functions of these MESs are unconfirmed. Future analysis of EAME1 and -2 may reveal the importance of these enzymes.

Finally, *ShMKS1* is known to be involved in the biosynthesis of methylketones in wild tomato [39]. Wild tomato plants are known to produce higher levels of methylketones than their domesticated relatives, which confers an advantage against herbivory [78,79]. Overexpression of methylketone biosynthesis pathway genes, including *ShMKS1* in Arabidopsis and tobacco, demonstrated heterologous production of methylketones but also led to severe growth defects [80]. Meanwhile, targeted expression in cultivated tomatoes showed only a slight increase in methylketone synthesis [80]. Further biological characterization of MES enzymes is crucial to clarify the diversity in roles served by this family.

## 5. MES Family Evolution in Land Plants

Though the MES family has been known for over two decades, it has received relatively less attention than some other protein families. Currently, all characterized members are from a small number of angiosperms, limiting the ability to study the evolution of this family. The SABATH family, which serves as the inverse of MES, has three times as many characterized members, including several from gymnosperms and non-seed plants [81,82,83,84]. This allows for greater understanding of the family and how it arose and evolved across plant taxa [4]. To gain better insights into this evolution for the MES family, a phylogenetic analysis was performed using bioinformatically identified *MES* genes from selected sequenced plants. The findings from this analysis can help bring attention to the importance of this family across plant lineages and demonstrate the origins of MES enzymes.

### 5.1. MES Phylogenetic Analysis

A total of twenty-three species were selected for MES identification and phylogenetic analysis, including two green algae (*Mesotaenium endlicherianum* and *Spirogloea muscicola*), three mosses (*Ceratodon purpureus*, *Physcomitrella patens*, and *Sphagnum fallax*), two liverworts (*Marchantia polymorpha* and *Ricciocarpos natans*), two hornworts (*Anthoceros agrestis* and *Anthoceros angustus*), two lycophytes (*Diphasiastrum complanatum* and *Selaginella moellendorffii*), four ferns (*Adiantum capillus-veneris*, *Alsophila spinulosa*, *Ceratopteris richardii*, and *Marsilea vestita*), two gymnosperms (*Picea abies* and *Thuja plicata*), and six angiosperms (*Amborella trichopoda*, *Arabidopsis thaliana*, *Nymphaea colorata*, *Oryza sativa*, *Populus trichocarpa*, and *Zea mays*). Annotated protein sequences of the 23 species were retrieved from public databases, including NCBI (https://www.ncbi.nlm.nih.gov, accessed on 1 September 2024) and Phytozome (https://phytozome-next.jgi.doe.gov, accessed on 1 September 2024), and searched using BLASTp [85] with known MESs (Table 1) as queries. A total of 448 MES genes were identified. Most notably, no MES members were identified in either algal species, indicating the family may have evolved specifically in embryophytes or may be limited to only certain algae. All 21 selected land plant species exhibit MES members ranging from three to twenty-eight per species. There is no obvious trend of family expansion, as most taxa represent both the low and high ends of this range in different species. This indicates that MES enzymes have continued to duplicate and serve important purposes in all lineages. Of the 448 MES proteins, 274 have an annotated protein length of 200 to 400 amino acids, which were used in subsequent phylogenetic analysis.

**Figure 3:**
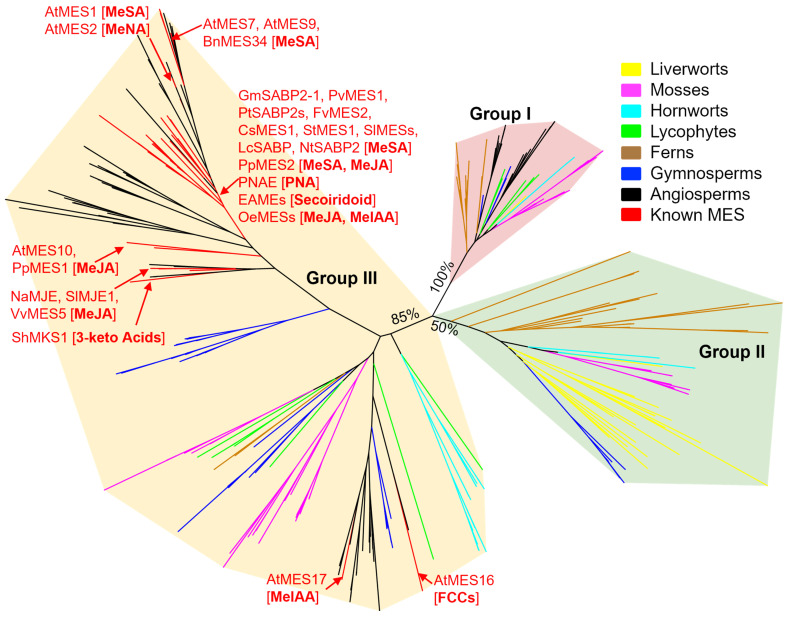
Phylogenetic analysis of MES proteins from 21 genomes representing various land plant taxa, plus all characterized *MESs*. Branches are color-coded as follows: liverworts, yellow; mosses, magenta; hornworts, cyan; lycophytes, green; ferns, brown; gymnosperms, blue; angiosperms, black; known MESs, red. Characterized MES members are indicated as red labels, with their substrates in brackets. The phylogenetic tree was constructed with RAxML (version 1.1.0) using the best-fit model LG + G4 + F with 1000 bootstrap replicates. Bootstrap support for the major groups is indicated. The tree was visualized and further annotated using iTOL (version 6.9.1).

Phylogenetic analysis of the 274 putative MES proteins identified from selected plants and the 32 functionally characterized MES proteins from flowering plants was performed. Different schemes of grouping (e.g., three groups or five groups) of MES proteins in the phylogenetic tree could be proposed. In this article, we preferred the clustering of three groups (denoted Groups I, II, and III) (Figure 3) with the consideration of the phylogeny of the major lineages of land plants. Group I, with a robust 100% bootstrap value, contains 66 putative MES members representing all 21 selected plant genomes, making it the most well-conserved MES clade in land plants. Group II, with a bootstrap value of 50%, contains 65 putative MES members from 13 selected plant genomes, but is absent in the surveyed lyiccophytes and angiosperms. Group III, with a strong bootstrap value of 85%, contains 143 putative MES members across 19 of the 21 surveyed plant genomes, being absent only in liverworts. The lack of putative MES members from selected plants in Groups II and III indicates potential gene loss in specific lineages, including liverworts, lycophytes, and angiosperms.

The presence of mosses, hornworts, ferns, and gymnosperms in all three MES groups—each with multiple genes per species—suggests that the MES family underwent significant duplication and diversification early in the evolution of land plants, prior to the divergence of these groups (Figure 3). This broad distribution across different lineages indicates that the core MES functions were established early and may have been conserved throughout evolutionary history. In contrast, liverworts are represented in Groups I and II but are absent in Group III. It has been previously hypothesized that liverworts diverged earlier than mosses and hornworts [86]. If this were the case, it could imply that the MES family originally consisted of two genes, with a third evolving after the divergence of liverworts. However, this model of land plant evolution is highly debatable, and many alternative hypotheses exist [86]. For example, it could be the case that there were three ancestral *MES* genes, but Group III was later lost in liverworts. Notably, the loss of Group II in lycophytes and angiosperms—as well as the potential loss of Group III in liverworts—suggests that some *MES* genes may have become redundant or non-essential in certain lineages, indicating functional divergence that occurred during MES evolution. Exploring the evolution of known MES functions can provide key insights into the origins of this enzyme family.

### 5.2. Enzymatic Evolution of MES Functions

Many enzyme families are proposed to arise through catalytic promiscuity, wherein ancestral enzymes had the ability to utilize noncanonical substrates, which are sometimes positively selected for and later become preferred substrates [87]. This mechanism of enzymatic evolution has been demonstrated in many families, including several belonging to the α/β hydrolase superfamily [88,89,90]. The hydroxynitrile lyases (HNLs), which were once grouped together with MES under CXE class II, are now thought to have diverged from MES enzymes around 100 million years ago through promiscuity [90]. NtSABP2 was shown to switch from esterase to HNL activity following only a two-amino-acid alteration to its primary sequence [91]. Further supporting this, AtMJE was demonstrated to have HNL activity in its native form, suggesting that even modern enzymes can exhibit promiscuity [92]. Based on the current understanding of catalytic evolution, it is expected that ancestral enzymes would have demonstrated more promiscuity than their modern counterparts [87]. It therefore stands to reason that the MES ancestors may have had a core functionality for essential plant functions, while also demonstrating alternative substrate utilization that would have later led to the evolution of new enzymes through duplication and divergence. With this in mind, it is useful to explore modern functions of MES enzymes and make inferences as to how these functions may have evolved from ancestral enzymes.

All characterized MES proteins fall into Group III, with the majority of characterized members clustering together in the largest angiosperm subgroup, including MeSA esterases, MJEs, PNAE, ShMKS1, and others (Figure 3). It appears that these functions arose from an expansion in angiosperms and gymnosperms, which is not seen in other lineages. This could mean these functions are unique to these lineages or that unrelated enzymes could serve these roles in other taxa. It is known that SA-mediated defense signaling extends to bryophytes, such as the moss *Physcomitrella patens* [93]. There has also been one bryophyte *SAMT* gene identified, namely *CsSAMT* from the liverwort *Conocephalum salebrosum* [84]. This indicates that methylation of SA takes place in liverworts and possibly other bryophytes, suggesting a need for an esterase to demethylate MeSA back to its active form. However, if such an enzyme exists, it appears not to be closely related to known MeSA esterases from angiosperms.

Similarly, it is not well understood to what extent JA and MeJA are involved in signaling processes in nonvascular plants. One recent study in the liverwort *Marchantia polymorpha* found that it contained at least some of the core components for the JA signaling pathway [94]. On the other hand, it is known that 12-oxo-phytodienoic acid (OPDA), the precursor to JA, can act in place of JA as a signaling molecule [95]. This has been shown to take place in *P. patens*, where OPDA was shown to respond to fungal infection and wounding [96,97]. If OPDA is used by bryophytes in place of JA, it is unclear if an *MES* gene would be required for this function. Further study of JA signaling in non-angiosperm plants is required to clarify the need for MJEs in other lineages.

The only characterized MES members that lie outside the aforementioned subgroup—but still within Group III—are AtMES16 and AtMES17. As previously discussed, AtMES16 serves to break down chlorophyll during leaf senescence [38,43]. Because of the importance of chlorophyll to all plants, it is sensible that regulating it through degradation would be an early-evolved plant function. However, as mentioned previously, it is unclear how well-conserved chlorophyll degradation pathways are across plant taxa [38]. The other outlier, AtMES17, is an esterase of MeIAA [9]. IAA is known to be crucial for growth and development throughout plants, including mosses and liverworts [98]. MeIAA is known in many plants as a reversible storage form and one of the ways that auxin levels are regulated [99]. However, it is unknown if MeIAA is a storage form utilized within nonvascular plants and therefore unclear if they would require an MeIAA esterase. There are many unknown MES members from across taxa contained within Group III, clustering with AtMES16 and AtMES17. Some of these may be homologs of these known members, but others may serve different functions. Overall, this subgroup appears to be an ancestral branch to the angiosperm group containing all other characterized MESs. This indicates that AtMES16 and AtMES17 could represent earlier functions in MES evolution. Notably, several MES enzymes demonstrate in vitro activity with MeIAA at high concentrations [12,29,36]. This could be representative of their ancestral activities, which have been mostly lost but still function under specific conditions. Further research on the members within this group can elucidate their functions and better our understanding of MES evolution.

While there is much to be discovered regarding Group III, there is even more work to be done in Groups I and II (Figure 3). These groups have no characterized MES members, leaving great doubt as to their biological and enzymatic functions. Members of Group II are found in all taxa except for lycophytes and angiosperms. It appears that this is a well-conserved group dating back to the origin of land plants, but it was somehow lost during the evolution of lycophytes and flowering plants. It could be that these members serve functions that are no longer needed in these lineages. Alternatively, it may be that these functions are served by more distantly related members, such as those in the expanded angiosperm subgroup within Group III. Group I, meanwhile, is represented in all taxa of land plants, and thus may be expected to serve a fundamental role. This could be an MES role that arose early in evolutionary history, and therefore understanding the functions within this group may hint at ancestral MES functions. Another possibility that must be considered is that Groups I and II may not be true MES enzymes at all. As mentioned previously, it is likely that the MES family evolved from other enzymes through catalytic promiscuity [87,90]. Members in Groups I and II, therefore, could represent ancestral roles from which MESs later evolved, or alternatively could be separate branches that evolved from the same ancestor as the MES family, leading to enzymatically unrelated functions. Because there are no characterized members within these groups, it is impossible to say whether they are true MES enzymes. It is apparent that focusing on a subset of enzyme activities, as well as limiting analysis to angiosperms, has hindered a broader understanding of this family. Further study of enzymes within these unknown groups is crucial for the understanding of the family as a whole and could lead to the discovery of new functions or even new gene families.

## 6. Conclusions and Future Directions

The origin and diversification of land plants were enabled by vast genetic innovations, among which are the ability of land plants to produce diverse metabolites and regulate their concentrations [100,101]. One example is the biosynthesis of diverse methyl esters of carboxylic acids by SABATH methyltransferases [4]. It is intriguing that land plants have also evolved enzymes—namely methylesterases (MESs)—to catalyze the reverse reaction: demethylation of methyl esters to convert them back to carboxylic acids. A number of conclusions can be drawn based on the current understanding of this family. First, *MESs* form a small gene family within land plants, as discussed in Section 5.1. Second, the majority of known MES enzymes catalyze demethylation of carboxyl methyl esters, with exceptions. Some members have evolved new catalytic activities, e.g., the decarboxylation activity of ShMKS1 [39]. Third, *MES* genes appear to be involved in diverse biological processes, ranging from phytohormone regulation to the biosynthesis of secondary metabolites (Figure 2). Fourth, *MES* genes appear to be specific to land plants, suggesting their origin in the common ancestor of land plants after the divergence from green algae. Phylogenetic analysis also implies that the common ancestor of land plants contained either two or three copies of *MES* genes, leading to the three extant groups seen today (Figure 3).

While significant progress has been made in our understanding of the MES family in the last two decades, much remains to be uncovered. So far, only a small number of *MES* genes from a very limited number of plant species have been studied (Table 1). Furthermore, within the present phylogenetic analysis, all functionally characterized members are in Group III (Figure 3). It will be highly informative to determine biochemical and biological functions of the members from Groups I and II. Members of Group I appear to be conserved across taxa. It is therefore interesting to ask whether the *MES* genes in this group have conserved functions. It will also be useful to look at known methylated products in consideration of possible MES substrates. For example, the SABATH methyltransferase family has nearly 20 known substrates, with only three of these currently being represented by respective MES enzymes [4]. Focusing on these unrepresented substrates could help identify new MES functions, particularly for substrates that are known to be demethylated for proper function. It may also be the case that some MES members have evolved new roles, not directly related to esterase activity. This is already seen with ShMKS1 and is likely to be true for some other members as well, particularly the more divergent they are from known enzymes. If similar instances exist for other MESs, alternative methodologies and substrate analysis will be required to identify their activity. Functional elucidation of the new members of the MES family in Groups I and II will also establish a proper context to address the question of functional evolution in the MES family. Novel catalytic activities may inspire new structural studies, as three-dimensional structures and reaction mechanisms have been solved for only a few members of the MES family [12,44,102,103]. As more MES structures become available, in silico tools can be employed to guide further research. Software such as AlphaFold3 can predict MES structures and functions based on known members, allowing for deeper insights into family evolution [104,105]. AlphaFold can also be used to redesign enzymes with altered activity [106,107], permitting researchers to leverage the natural diversity in the MES family. As additional MES functions become known, the extent of this engineering potential will expand as well. The MES family is already known to be important in defense and development processes. Continuing to identify novel members of this family and characterize their functions will further highlight its importance and help discern its evolutionary origins.

## Figures and Tables

**Table 1:** List of all biochemically characterized MES enzymes with known substrates.

Enzyme	Species	Major Substrate ^a^	Reference
AtMES1	*Arabidopsis thaliana*	MeSA	Vlot et al. 2008 [22]
AtMES7	*Arabidopsis thaliana*	MeSA	Gao et al. 2021 [23]
AtMES9	*Arabidopsis thaliana*	MeSA	Vlot et al. 2008 [22]
BnMES34	*Brassica napus*	MeSA	Jia et al. 2024a [24]
CsMES1	*Citrus sinensis*	MeSA	Lima Silva et al. 2019 [25]
FvMES2	*Fragaria vesca*	MeSA	Jia et al. 2024b [26]
GmSABP2-1	*Glycine max*	MeSA	Lin et al. 2024 [27]
LcSABP	*Lycium chinense*	MeSA	Li et al. 2019 [28]
PtSABP2-1	*Populus trichocarpa*	MeSA	Zhao et al. 2009 [29]
PtSABP2-2	*Populus trichocarpa*	MeSA	Zhao et al. 2009 [29]
PvMES1	*Phaseolus vulgaris*	MeSA	Xue et al. 2021 [30]
NtSABP2	*Nicotiana tabacum*	MeSA	Forouhar et al. 2005 [12]
SlMES1	*Solanum lycopersicum*	MeSA	Frick et al. 2023 [31]
SlMES2	*Solanum lycopersicum*	MeSA	Frick et al. 2023 [31]
SlMES3	*Solanum lycopersicum*	MeSA	Frick et al. 2023 [31]
SlMES4	*Solanum lycopersicum*	MeSA	Frick et al. 2023 [31]
StMES1	*Solanum tuberosum*	MeSA	Manosalva et al. 2010 [32]
PpMES2	*Prunus persica*	MeSA/MeJA	Cao et al. 2019 [18]
AtMES10	*Arabidopsis thaliana*	MeJA	Koo et al. 2013 [33]
PpMES1	*Prunus persica*	MeJA	Cao et al. 2019 [18]
SlMJE1	*Solanum lycopersicum*	MeJA	Stuhlfelder et al. 2004 [11]
NaMJE	*Nicotiana attenuata*	MeJA	Wu et al. 2008 [34]
VvMES5	*Vitis vinifera*	MeJA	Zhao et al. 2016 [35]
OeMES1	*Olea europaea*	MeJA, MeIAA	Volk et al. 2019 [36]
OeMES2	*Olea europaea*	MeJA, MeIAA	Volk et al. 2019 [36]
AtMES17	*Arabidopsis thaliana*	MeIAA	Yang et al. 2008 [9]
AtMES2	*Arabidopsis thaliana*	MeNA	Wu et al. 2018 [37]
AtMES16	*Arabidopsis thaliana*	FCCs	Crist et al. 2012 [38]
ShMKS1	*Solanum habrochaites*	3-keto acids	Fridman et al. 2005 [39]
PNAE	*Rauvolfia serpentina*	PNA	Dogru et al. 2000 [10]
EAME1	*Olea europaea*	Secoiridoids	Volk et al. 2019 [36]
EAME2	*Olea europaea*	Secoiridoids	Volk et al. 2019 [36]

^a^ methyl salicylate (MeSA), methyl jasmonate (MeJA), methyl indole-3-acetate (MeIAA), methyl nicotinate (MeNA), fluorescent chlorophyll catabolites (FCCs), 3-keto acids, polyneuridine aldehyde (PNA), secoiridoids.

## Data Availability

The original contributions presented in the study are included in the article. Further inquiries may be directed to the corresponding author.
